# Supplementary material for: A multi-omic meta-analysis reveals novel mechanisms of insecticide resistance in malaria vectors
Source: Commun Biol. 2025 May 23;8:790. doi: 10.1038/s42003-025-08221-6 (PMC12102355; doi:10.1038/s42003-025-08221-6)
Supplement: Supplementary file 1 — Supplementary Information [file 42003_2025_8221_MOESM1_ESM.pdf]

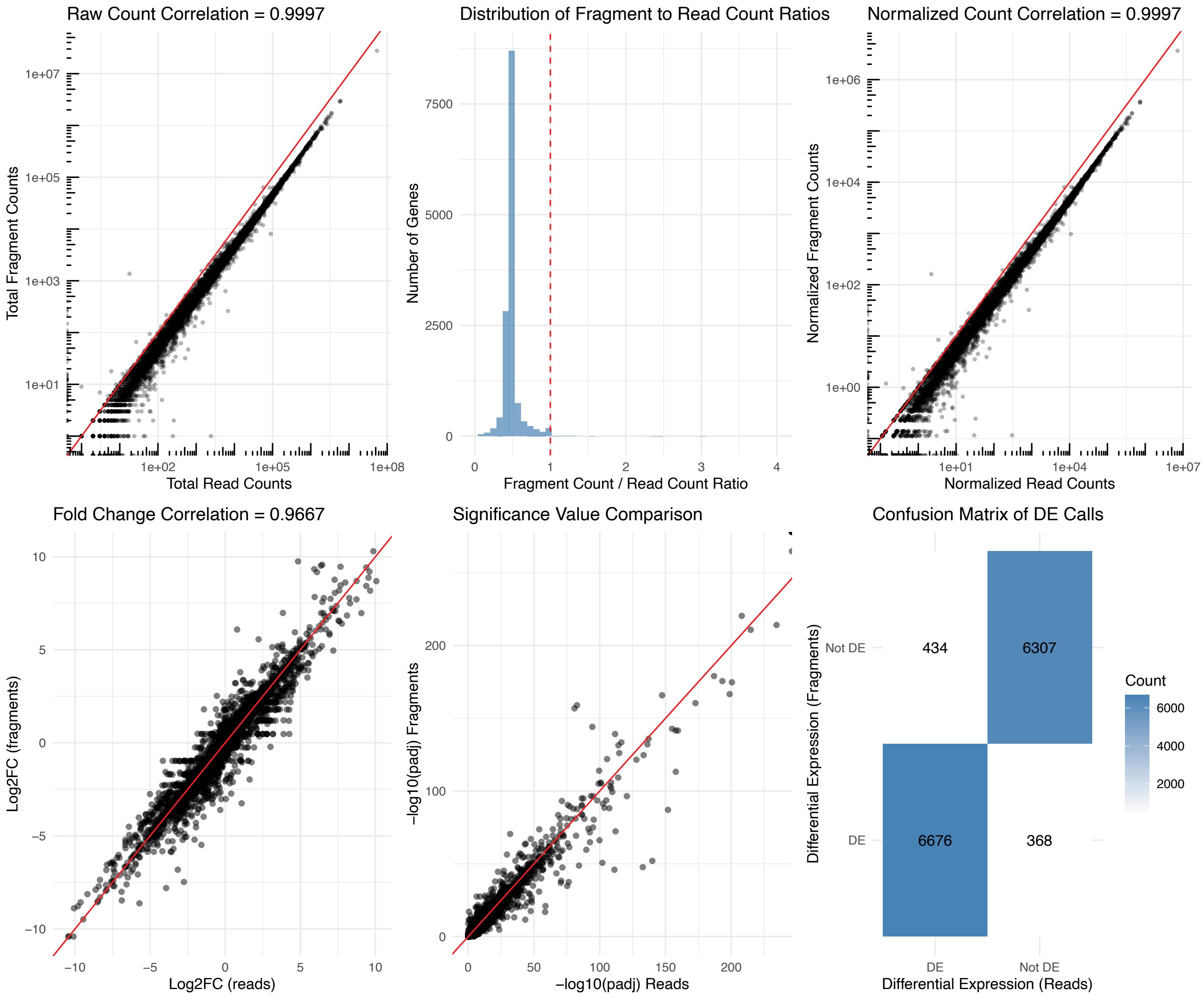

Supplementary Figure 1: Correlation between fragment and read counts. A. Correlation of total fragment (y-axis) and read counts (x-axis). B. The ratio of fragments:reads (x-axis) is expected to be around 0.5, majority of genes (y-axis) show this relationship. C. Normalised fragments counts (y-axis) compared to normalised read counts (x-axis). D. Calculated log2 fold change for fragments (y-axis) and counts (x-axis) utilising the pipeline used in this manuscript. E. As in D but for p-values. F. Shows the number of differentially expressed genes for fragments (y-axis) and reads (x-asix). DE = differential, Not DE = not differential with a padj < 0.05. In A, C, D and E the red line is y=x, correlation is shown above the graph, where relevant.

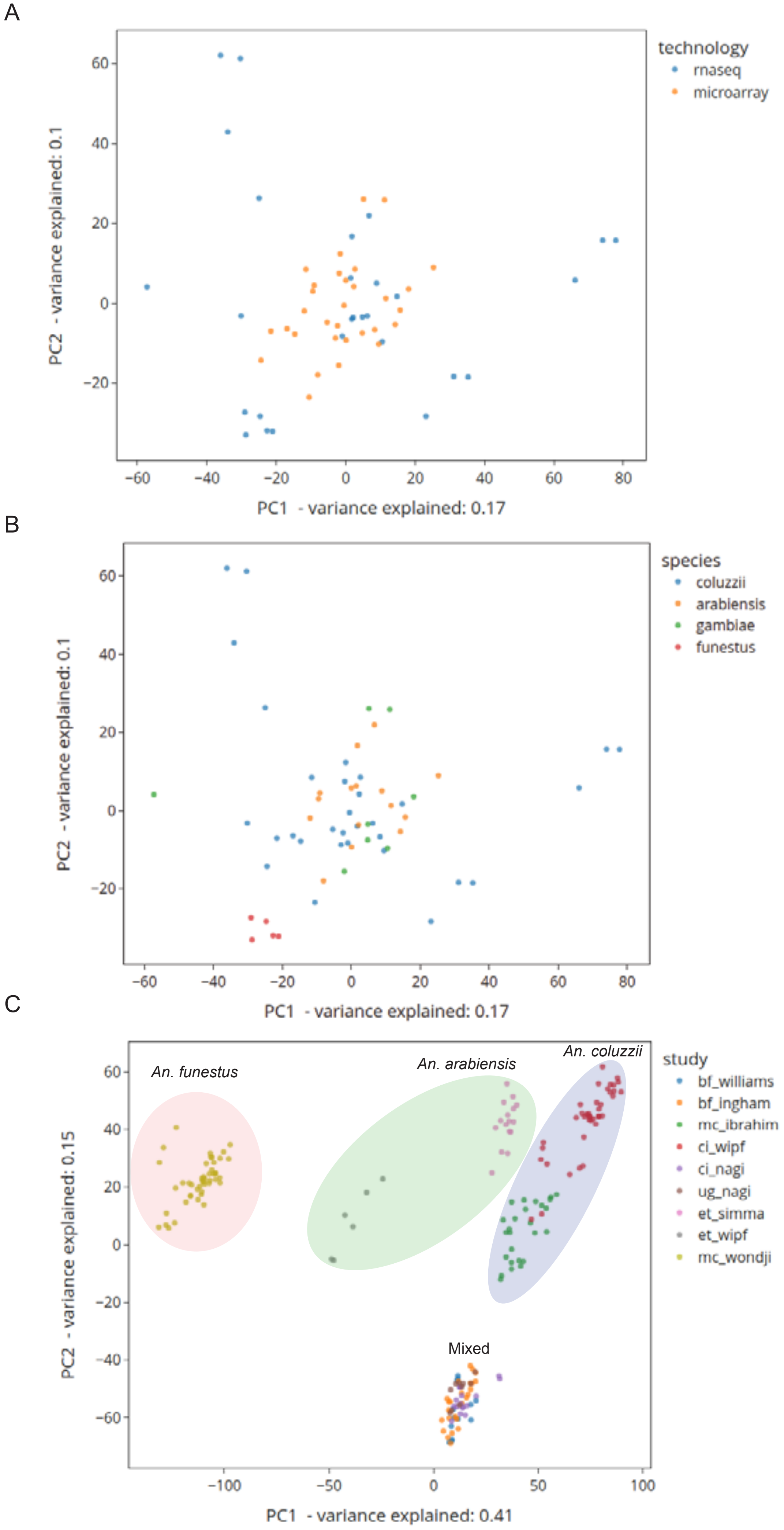

Supplementary Figure 2: PCA analyses. A. PCA analysis coloured by technology of the combined RNAseq data from this study and collated microarray data from Ingham et al. 2018. B. As in A but coloured by species. C. PCA analysis of the RNAseq data coloured by study. Species are illustrated by ellipses and/or text descriptions. In each the ratio of variance explained is shown next to the principal component displayed.

Supplementary Table 1: Metadata from AnoExpress Datasets. The title of the RNAseq comparison as seen on AnoExpress indicating the resistant population and susceptible comparator, the resistant and susceptible populations, associated species, country and location of collection of the resistant populations, as well as the reference for each experiment.

RNAseq Comparison Name	Resistant Population	Susceptible Population	Species	Country	Lattitude	Longitude	Reference
Gou_v_Moz	Gaoua	Moz	arabiensis	Burkina Faso	10.533	-3.283	https://doi.org/10.3390/insects13030247
Asendabo_v_Moz	Asendabo	Moz	arabiensis	Ethiopia	7.7728	37.22979	https://doi.org/10.1002/ps.5374
Chewaka_v_Moz	Chewaka	Moz	arabiensis	Ethiopia	8.89366	36.15594	https://doi.org/10.1002/ps.5374
Tolay_v_Moz	Tolay	Moz	arabiensis	Ethiopia	8.356	37.3409	https://doi.org/10.1002/ps.5374
Ethiopia_v_Dongola	Ethiopia	Dongola	arabiensis	Ethiopia	7.7728	37.22979	https://doi.org/10.1016/j.ibmb.2021.103655
Tiefora_v_Ngousso	Tiefora	Ngousso	coluzzii	Burkina Faso	11.41	-4.41	https://doi.org/10.3390/insects13030247
Ban_v_BanS	Banfora-Original	BanS	coluzzii	Burkina Faso	10.6333	-4.563	https://doi.org/10.1371/journal.pgen.1009970
BanRe_v_BanS	Banfora-Reselected	BanS	coluzzii	Burkina Faso	10.6333	-4.563	https://doi.org/10.1371/journal.pgen.1009970
VK7_v_Kisumu	VK7	Kisumu	coluzzii	Burkina Faso	11.4117	-4.36277	https://doi.org/10.3390/insects13030247
Cameroon_v_Ngousso	Cameroon	Ngousso	coluzzii	Cameroon	10.844	14.9	https://doi.org/10.1101/2022.03.21.485146
Chad_v_Ngousso	Chad	Ngousso	coluzzii	Chad	12.1	15.03	https://doi.org/10.1101/2022.03.21.485146
Niger_v_Ngousso	Niger	Ngousso	coluzzii	Niger	13.7	7.98	https://doi.org/10.1101/2022.03.21.485146
Nigeria_v_Ngousso	Nigeria	Ngousso	coluzzii	Nigeria	9.9875	12.3605	https://doi.org/10.1101/2022.03.21.485146
Agboville_v_Mali	Agboville	Mali	coluzzii	Cote d'Ivoire	5.9448	-4.21468	https://doi.org/10.1371/journal.pgen.1009963
Agboville_v_Ngousso	Agboville	Ngousso	coluzzii	Cote d'Ivoire	5.9448	-4.21468	https://doi.org/10.1371/journal.pgen.1009963
Dabou_v_Mali	Dabou	Mali	coluzzii	Cote d'Ivoire	5.331	-4.372	https://doi.org/10.1371/journal.pgen.1009963
Dabou_v_Ngousso	Dabou	Ngousso	coluzzii	Cote d'Ivoire	5.331	-4.372	https://doi.org/10.1371/journal.pgen.1009963
Tiassale_v_Mali	Tiassale	Mali	coluzzii	Cote d'Ivoire	5.9006	-4.835	https://doi.org/10.1371/journal.pgen.1009963
Tiassale_v_Ngousso	Tiassale	Ngousso	coluzzii	Cote d'Ivoire	5.9006	-4.835	https://doi.org/10.1371/journal.pgen.1009963
Bouake_colu_unexp_v_Ngousso	Bouake_colu	Ngousso	coluzzii	Cote d'Ivoire	7.73167	-5.0001	https://zenodo.org/doi/10.5281/zenodo.12750269
Cam_fun_v_Fang	Cameroon_fun	Fang	funestus	Cameroon	6.08	11.47	https://doi.org/10.3390/genes12040561
Fumoz_v_Fang	Fumoz	Fang	funestus	Mozambique	-22.77488	33.809	https://doi.org/10.3390/genes12040561
Ghana_fun_v_Fang	Ghana_fun	Fang	funestus	Ghana	5.933	-1.6	https://doi.org/10.3390/genes12040561
Malawi_fun_v_Fang	Malawi_fun	Fang	funestus	Malawi	-16.01	34.7833	https://doi.org/10.3390/genes12040561
Uganda_fun_v_Fang	Uganda_fun	Fang	funestus	Uganda	0.677	34.184	https://doi.org/10.3390/genes12040561
Bak_v_Kisumu	Bakaridjan	Kisumu	gambiae	Burkina Faso	10.407	-4.563	https://doi.org/10.3390/insects13030247
Bouake_gamb_unexp_v_Kisumu	Bouake_gamb	Kisumu	gambiae	Cote d'Ivoire	7.73167	-5.0001	https://zenodo.org/doi/10.5281/zenodo.12750269
BusiaSurvivors_v_Kisumu	Busia	Kisumu	gambiae	Uganda	0.47648	34.09281	https://doi.org/10.1111/1755-0998.13759

Supplementary Table 2: Pearson's correlation coefficient between different count generations.

	hisat2-ftcounts	kallisto	hisat2-htseqcount
**hisat2-ftcounts**	1	094388007	0992229027
**kallisto**	094388007	1	0922069014
**hisat2-htseqcount**	0992229027	0922069014	1

Supplementary Table 3: Candidate insecticide resistance genes. These genes show a median fold change of greater than 2, and median counts of greater than 5. Table shows Gene identifier, name and description from VectorBase and the associated log2 median and mean fold changes.

GeneID	GeneName	GeneDescription	median log2 Fold Change	median Fold Change
AGAP001684		Alkaline phosphatase [Source:UniProtKB/TrEMBL;Acc:Q7PY02]	2.77	6.82
AGAP008447	CPLCG4	cuticular protein CPLCG family (CPLCG4) [Source:VB Community Annotation]	2.57	5.94
AGAP006417		venom allergen [Source:VB Community Annotation]	2.51	5.7
AGAP002867	CYP6P4	cytochrome P450 [Source:VB Community Annotation]	2.51	5.7
AGAP002865	CYP6P3	cytochrome P450 [Source:VB Community Annotation]	2.41	5.31
AGAP000818	CYP9K1	cytochrome P450 [Source:VB Community Annotation]	2.21	4.63
AGAP000820	CPR125	cuticular protein RR-2 family 125 [Source:VB Community Annotation]	2.08	4.23
AGAP006868	CPR140	cuticular protein RR-1 family 140 [Source:VB Community Annotation]	2.08	4.23
AGAP008212	CYP6M2	cytochrome P450 [Source:VB Community Annotation]	2.05	4.14
AGAP009758	CPLCP11	cuticular protein CPLCP11 [Source:VB Community Annotation]	2.02	4.06
AGAP009194	GSTE2	glutathione S-transferase epsilon class 2 [Source:VB Community Annotation]	1.83	3.56
AGAP002866	CYP6P5	cytochrome P450 [Source:VB Community Annotation]	1.75	3.36
AGAP006222		glucosyl/glucuronosyl transferases [Source:VB Community Annotation]	1.73	3.32
AGAP002864	CYP6P15P	cytochrome P450 [Source:VB Community Annotation]	1.69	3.23
AGAP010830	TEP9	thioester-containing protein 9 [Source:VB Community Annotation]	1.63	3.1
AGAP005145			1.51	2.85
AGAP009759	CPLCP12	cuticular protein CPLCP12 [Source:VB Community Annotation]	1.47	2.77
AGAP003626			1.45	2.73
AGAP003257	GSTU2	glutathione S-transferase unclassified 2 [Source:VB Community Annotation]	1.42	2.68
AGAP010831	TEP8	thioester-containing protein 8 [Source:VB Community Annotation]	1.42	2.68
AGAP008648			1.38	2.6
AGAP011477		eupolytin [Source:VB Community Annotation]	1.34	2.53
AGAP004690	CPF3	cuticular protein 3 from fifty-one aa family [Source:VB Community Annotation]	1.32	2.5
AGAP005194			1.25	2.38
AGAP004455	GNBPB1	3-glucan binding protein [Source:VB Community Annotation]	1.2	2.3
AGAP002863	COEAE6O	carboxylesterase alpha esterase [Source:VB Community Annotation]	1.17	2.25
AGAP009766			1.15	2.22
AGAP008450			1.12	2.17
AGAP006223		glucosyl/glucuronosyl transferases [Source:VB Community Annotation]	1.09	2.13
AGAP004730		phospholipase A2, venom [Source:VB Community Annotation]	1.08	2.11
AGAP009946	GSTMS3	glutathione transferase microsomal 3 [Source:VB Community Annotation]	1.06	2.08
AGAP011431			1.05	2.07
AGAP001676		actin, cytoplasmic [Source:VB Community Annotation]	1.03	2.04
AGAP002862	CYP6AA1	cytochrome P450 [Source:VB Community Annotation]	1.02	2.03

Supplementary Table 4: Enrichment analysis on the top 5% median over expressed genes, showing significant terms of either GO Terms or PFAM

annotation	pval	padj	descriptions	Type
GO:0042302	2.35407E-27	1.11512E-23	structural constituent of cuticle	GO Term
GO:0016705	3.3909E-19	8.03134E-16	oxidoreductase activity, acting on paired donors, with incorporation or reduction of molecular oxygen	GO Term
GO:0005506	3.01355E-18	4.7584E-15	iron ion binding	GO Term
GO:0020037	5.74861E-17	6.80779E-14	heme binding	GO Term
GO:0004497	9.5308E-17	9.02948E-14	onooxygenase activity	GO Term
GO:0005576	5.84648E-15	4.6158E-12	xtracellular region	GO Term
GO:0004252	1.03376E-12	6.99557E-10	serine-type endopeptidase activity	GO Term
GO:0008061	3.92804E-12	2.32589E-09	chitin binding	GO Term
GO:0006030	2.44974E-11	1.28938E-08	chitin metabolic process	GO Term
GO:0055114	3.82401E-11	1.81143E-08	obsolete oxidation-reduction process	GO Term
GO:0016491	1.1096E-10	4.77832E-08	oxidoreductase activity	GO Term
GO:0007608	3.50243E-09	1.38258E-06	sensory perception of smell	GO Term
GO:0031012	5.08306E-09	1.85219E-06	xtracellular matrix	GO Term
GO:0005549	7.11709E-09	2.40812E-06	odorant binding	GO Term
GO:0006508	1.26401E-08	3.99175E-06	proteolysis	GO Term
GO:0050911	8.67665E-07	0.000256883	detection of chemical stimulus involved in sensory perception of smell	GO Term
GO:0004984	1.04996E-06	0.000285974	olfactory receptor activity	GO Term
GO:0050896	1.08666E-06	0.000285974	response to stimulus	GO Term
GO:0008236	1.2908E-06	0.000321818	serine-type peptidase activity	GO Term
GO:0004364	4.96994E-06	0.001121076	glutathione transferase activity	GO Term
GO:0006749	4.96994E-06	0.001121076	glutathione metabolic process	GO Term
GO:0050909	8.2662E-05	0.017738431	sensory perception of taste	GO Term
GO:0008233	8.61271E-05	0.017738431	peptidase activity	GO Term
GO:0043025	0.000114694	0.022637729	uronal cell body	GO Term
GO:0008527	0.000226549	0.041275532	taste receptor activity	GO Term
GO:0050912	0.000226549	0.041275532	detection of chemical stimulus involved in sensory perception of taste	GO Term
GO:0102336	0.000327703	0.044238568	3-oxo-arachidoyl-CoA synthase activity	GO Term
GO:0034626	0.000327703	0.044238568	fatty acid elongation, polyunsaturated fatty acid	GO Term
GO:0009922	0.000327703	0.044238568	fatty acid elongase activity	GO Term
GO:0102337	0.000327703	0.044238568	3-oxo-cerotoyl-CoA synthase activity	GO Term
GO:0019367	0.000327703	0.044238568	fatty acid elongation, saturated fatty acid	GO Term
GO:0034625	0.000327703	0.044238568	fatty acid elongation, monounsaturated fatty acid	GO Term
GO:0102338	0.000327703	0.044238568	3-oxo-lignoceronyl-CoA synthase activity	GO Term
GO:0102756	0.000327703	0.044238568	very-long-chain 3-ketoacyl-CoA synthase activity	GO Term
GO:0008146	0.000327703	0.044238568	sulfotransferase activity	GO Term
GO:0016747	0.000336202	0.044238568	cyltransferase activity, transferring groups other than amino-acyl groups	GO Term
C_tripleX	1.65444E-37	6.11317E-34	NA	PFAM
Chitin_bind_4	1.75599E-23	3.24419E-20	NA	PFAM
CBM_14	1.0889E-21	1.34117E-18	NA	PFAM
p450	1.75137E-19	1.61783E-16	NA	PFAM
Trypsin	3.64858E-13	2.6963E-10	NA	PFAM
7tm_6	6.02906E-07	0.000371289	NA	PFAM
GST_N_3	3.97109E-06	0.002096168	NA	PFAM
GST_C	1.05601E-05	0.004877462	NA	PFAM
Mucin-like	8.84402E-05	0.029999981	NA	PFAM
CPCFC	8.84402E-05	0.029999981	NA	PFAM
Hemocyanin_N	0.000105548	0.029999981	NA	PFAM
Hemocyanin_M	0.000105548	0.029999981	NA	PFAM
Hemocyanin_C	0.000105548	0.029999981	NA	PFAM
7tm_7	0.000134316	0.035449869	NA	PFAM

Supplementary Table 5: Genes significantly differential in over 19 / 28 of the datasets. VectorBase ID, Gene Name, Gene Description and log2 fold changes for each experimental comparison. Top: up regulated, Bottom: Down regulated

GeneID	GeneName	GeneDescription	Tiefora_v_Ngousso	Gou_v_Moz	Ban_v_BanS	BanRe_v_BanS	Bak_v_Kisumu	VK7_v_Kisumu	Cameroon_v_Ngousso	Chad_v_Ngousso	Niger_v_Ngousso	Nigeria_v_Ngousso	Agboville_v_Mali	Agboville_v_Ngousso	Dabou_v_Mali	Dabou_v_Ngousso	Tiassale_v_Mali	Tiassale_v_Ngousso	Cam_fun_v_Fang	Fumoz_v_Fang	Ghana_fun_v_Fang	Malawi_fun_v_Fang	Uganda_fun_v_Fang	Asendabo_v_Moz	Chewaka_v_Moz	Tolay_v_Moz	Ethiopia_v_Dongola	Bouake_gamb_unexp_v_Kisumu	Bouake_colu_unexp_v_Ngousso	BusiaSurvivors_v_Kisumu
AGAP00	CYP6P3	cytochrome P450	4.26	1.07	1.36	-0.76	2.03	3.9	1.26	1.87	0.41	-0.32	3.82	5.98	4.07	6.22	4.28	6.45	1.46	5.19	3.58	5.44	2.61	2.41	4.7	2.85	0.47	1	5.19	-3.39
AGAP00	CYP6Z2	cytochrome P450	2.64	1.14	0.58	-2.17	2.55	4.33	3.46	1.54	2.07	1.5	1.35	2.11	0.46	1.2	0.34	1.1	-0.2	-0.02	0.52	0.55	0.45	1.27	1.98	0.7	-0.85	1.32	6.89	0.59
AGAP00	GSTD3	glutathione S-tran	2.05	1.05	1.44	0.39	-0.25	0.9	-0.39	0.67	0.18	1.22	2.09	1.26	2.63	1.79	2.03	1.21	0.83	1.24	1.92	1.79	1.38	1.72	1.31	1.71	1.75	0.03	0.96	0.26
AGAP010205			1.27	0.51	0.35	-1.54	1.51	0.32	0.01	0.25	1.21	1.22	-0.13	-0.24	0.52	0.39	0.96	0.85	-1.31	-1.04	-1.17	-1	-1.41	0.76	0.63	0.91	0.72	0.6	-0.51	1
AGAP00	CYP6Z3	cytochrome P450	2.81	-0.26	0.76	-1.56	1.54	2.36	2.25	0.75	1.52	1.6	1.35	2.11	0.46	1.2	0.34	1.1	-0.2	-0.02	0.52	0.55	0.45	1.27	1.98	0.7	-0.85	1.28	5.57	1.25
AGAP00	CYP6P4	cytochrome P450	2.05	-0.85	2.56	0.59	1.65	4.7	2.3	0.59	-0.28	1.11	3.89	5.32	3.4	4.82	4.16	5.58	1.08	5.16	3.49	5.41	2.51	2.51	3.12	3.14	2.03	0.36	3.58	-1.07
AGAP010534			0.95	-0.27	0.66	0.46	0.29	0.18	-0.46	-0.44	0.15	0.23	0.94	1.2	0.79	1.03	1.07	1.33	-0.46	-0.29	-0.51	-0.55	-0.78	1.2	0.84	1.39	0.26	0.05	0.51	-0.68
AGAP00	CYP9K1	cytochrome P450	0.64	0.2	1.08	-0.73	4.65	2	1.58	0.44	0.31	1.08	3.45	3.81	2.98	3.32	2.21	2.56	0	0.92	1.32	0.93	2.91	2.27	2.89	2.28	1.37	2.87	2.23	-0.39
AGAP00	CYP4H17	cytochrome P450	1.72	1.9	0.54	-0.09	3.89	2.46	2.08	2.18	1.47	3.15	1.73	2.94	1.68	2.88	1.81	3.02	0.33	0.8	1.18	0.85	0.68	1.77	2.52	2.21	1.14	2.3	2.71	0.44
AGAP02	CYP4H18	cytochrome P450	1.19	2.72	-1.95	-1.57	2.16	2.17	1.88	2.42	2.37	3.57	-2.32	0.82	-0.54	2.57	-2.48	0.66	0.33	0.8	1.18	0.85	0.68	2.49	1.78	2.73	0.46	2.69	1.82	3.21
AGAP00	GSTE2	glutathione S-tran	1.83	0.33	2.05	0.03	-0.98	2.55	3.8	2.87	2.66	3.06	2.44	-0.09	2.85	0.3	2.05	-0.47	2.13	1.32	2.94	1.1	1.63	2.13	0.82	3.14	1.32	0.3	0.21	-2.26
AGAP003483			-0.87	1.23	-1.94	-0.5	1.52	-0.85	-0.37	0.97	1.11	0.5	1.96	1.17	2.23	1.42	1.93	1.14	0.04	0.34	0.51	0.64	0.15	0.3	1.21	0.77	1.9	-1.06	-0.37	0.92
AGAP00	TRYP4	trypsin 4 [Source:	1.96	0.95	1.78	1.24	-1.9	-0.6	-1.15	-0.96	-3.07	-5.31	3.35	3.94	3.05	3.63	3.21	3.8	0.6	0.86	0.82	0.32	0.41	0.6	3.4	1.71	0.45	-2.28	0.18	-1.21
AGAP006222		glucosyl/glucuron	0.75	1.27	2.17	0.03	1.53	1.7	1.73	1.1	0.53	1.73	2.21	2.75	2.47	3	1.94	2.48	-0.58	0.03	0.71	0.29	-0.03	2.22	3.44	2.52	1.08	0.15	2.43	-2.83
AGAP013481			0.12	-0.01	0.41	0.47	0.34	0.06	-0.62	0.25	0.56	0.16	1.26	1.09	1.04	0.85	1.18	1.01	-0.74	-0.57	-0.74	-0.81	-1	1.02	1.13	1.2	2.64	0.47	0.29	-0.4
AGAP003357		RAG1-activating p	0.2	0.77	0.13	-0.64	0.36	0.37	-0.29	-0.58	-0.76	-0.56	1.94	1.96	1.99	2.01	1.9	1.93	0.81	1.25	0.97	1.47	1.02	0.59	0.98	1.11	0.35	-0.07	1.13	-0.39
AGAP00	CYP6M2	cytochrome P450	1	-0.46	3.18	0.79	2.01	3.25	0.51	0.08	-1.39	-0.01	3.38	4.63	3.05	4.28	2.75	4	0.27	0.47	0.82	0.8	0.56	3.2	4.27	3.12	1.73	0.67	2.05	-1.84
AGAP00	CYP6P5	cytochrome P450	0.51	1.53	0.58	-1.1	2.27	3.02	-1.41	-2.77	-4.1	-1.76	4.71	4.67	4.64	4.6	3.98	3.95	1.34	4.86	3.3	5.11	2.37	1.75	2.89	2.2	-2.06	-1.93	2.45	-1.66

GeneID	GeneName	GeneDescription	Tiefora_v_Ngousso	Gou_v_Moz	Ban_v_BanS	BanRe_v_BanS	Bak_v_Kisumu	VK7_v_Kisumu	Cameroon_v_Ngousso	Chad_v_Ngousso	Niger_v_Ngousso	Nigeria_v_Ngousso	Agboville_v_Mali	Agboville_v_Ngousso	Dabou_v_Mali	Dabou_v_Ngousso	Tiassale_v_Mali	Tiassale_v_Ngousso	Cam_fun_v_Fang	Fumoz_v_Fang	Ghana_fun_v_Fang	Malawi_fun_v_Fang	Uganda_fun_v_Fang	Asendabo_v_Moz	Chewaka_v_Moz	Tolay_v_Moz	Ethiopia_v_Dongola	Bouake_gamb_unexp_v_Kisumu	Bouake_colu_unexp_v_Ngousso	BusiaSurvivors_v_Kisumu
AGAP006961		molecular chaper	-1.51	-0.83	-0.53	1.19	-0.33	-1.58	-1.05	-1.09	-0.98	-0.74	-0.3	-0.2	-0.47	-0.38	-0.01	0.09	-1.58	-1.6	-2.02	-1.93	-2.12	1.23	0.88	0.47	-0.6	-2.93	-1.01	-2.95
AGAP01	GPROP4	long wavelength s	0.87	-0.33	0.52	-1.15	-0.24	-0.47	0.83	-0.82	-0.4	-0.11	-0.4	1.13	-0.54	0.98	0.49	2.03	-0.85	-0.8	-0.9	-0.95	-0.92	-0.78	-0.87	-0.89	-0.09	0.18	0.89	-1.19
AGAP00	GPROP6	long wavelength s	-0.99	0.25	0.68	-2.19	-0.04	-0.85	-1.45	-2.69	-2.07	-1.35	-0.4	1.13	-0.54	0.98	0.49	2.03	-0.85	-0.8	-0.9	-0.95	-0.92	-0.78	-0.87	-0.89	-0.09	1.88	-2.03	-1.37
AGAP013311			-1.67	0.72	-1.22	-0.83	0.23	-0.81	-1.42	-2.36	-1.39	-1.29	0.61	0.26	1.9	1.54	1.95	1.61	-1.9	-1.52	-1.91	-2.17	-2.03	-0.8	-0.16	-1.18	-0.52	-0.62	-1.12	-3.02
AGAP002461			-0.44	-0.25	-0.58	1.04	-0.7	0.45	-0.78	-0.64	-0.46	-0.76	-0.48	-0.73	-0.65	-0.92	-1.08	-1.33	-0.8	-0.53	-0.98	-1.02	-0.67	2.13	0.7	1.11	0.32	-0.4	1.11	-0.56
AGAP009991		Cyclophilin B prec	-0.28	1.41	-0.23	-1.57	0.26	-0.96	-0.89	-1.49	-0.94	-0.74	-1.9	0.92	-1.81	0.99	-0.05	2.77	-0.74	-0.87	-1	-0.35	-0.56	-0.82	-0.64	-0.87	0.14	-0.3	-1.31	0.81
AGAP010427		serine palmitoyltr	-0.16	-0.33	0.19	0.52	-0.17	-0.18	-0.49	-0.31	-0.27	-0.49	-0.55	-0.75	-0.59	-0.8	-0.55	-0.74	-0.6	-0.15	-0.59	-0.58	-0.6	1.18	0.25	0.23	-0.24	-0.37	-0.04	0.22
AGAP001600		Ser/Thr protein pl	-0.1	-1.2	0.4	-0.64	0.57	0.95	0.11	-0.91	-0.96	-1.3	-0.15	-0.06	-0.24	-0.16	-0.42	-0.32	-1.06	-0.78	-1.07	-0.83	-1.24	-0.62	-0.57	-0.9	-0.95	0.69	0.9	0.41

Supplementary Table 6: Detoxification genes showing a mean or median fold change over 2. Headers show VectorBase ID, gene name, gene description, median log2 fold change, median fold change, mean log2 fold change and mean fold change.

GeneID	GeneName	GeneDescription	median log2 Fold Change	median Fold Change	mean log2 Fold Change	mean Fold Change
AGAP002865	CYP6P3	cytochrome P450 [Source:VB Community Annotation]	2.73	6.63	2.764642857	6.8
AGAP002867	CYP6P4	cytochrome P450 [Source:VB Community Annotation]	2.535	5.8	2.603928571	6.08
AGAP002894	CYP6Z4	cytochrome P450 [Source:VB Community Annotation]	2.315	4.98	1.630714286	3.1
AGAP002866	CYP6P5	cytochrome P450 [Source:VB Community Annotation]	2.235	4.71	1.569285714	2.97
AGAP000818	CYP9K1	cytochrome P450 [Source:VB Community Annotation]	1.79	3.46	1.756428571	3.38
AGAP008358	CYP4H17	cytochrome P450 [Source:VB Community Annotation]	1.79	3.46	1.796071429	3.47
AGAP009194	GSTE2	glutathione S-transferase epsilon class 2 [Source:VB Community Annotation]	1.73	3.32	1.430714286	2.7
AGAP028019	CYP4H18	cytochrome P450 [Source:VB Community Annotation]	1.485	2.8	1.1675	2.25
AGAP002864	CYP6P15P	cytochrome P450 [Source:VB Community Annotation]	1.475	2.78	1.439285714	2.71
AGAP006700	COEAE8O	carboxylesterase alpha esterase [Source:VB Community Annotation]	1.415	2.67	1.5125	2.85
AGAP008212	CYP6M2	cytochrome P450 [Source:VB Community Annotation]	1.365	2.58	1.684642857	3.21
AGAP004171	GSTD8	glutathione S-transferase delta class 8 [Source:VB Community Annotation]	1.305	2.47	1.129285714	2.19
AGAP004382	GSTD3	glutathione S-transferase delta class 3 [Source:VB Community Annotation]	1.25	2.38	1.184642857	2.27
AGAP010966	CYP6AJ1	cytochrome P450 [Source:VB Community Annotation]	1.235	2.35	1.2725	2.42
AGAP008218	CYP6Z2	cytochrome P450 [Source:VB Community Annotation]	1.17	2.25	1.335714286	2.52
AGAP010414	CYP4C28	cytochrome P450 [Source:VB Community Annotation]	1.17	2.25	0.974642857	1.97
AGAP009195	GSTE1	glutathione S-transferase epsilon class 1 [Source:VB Community Annotation]	1.16	2.23	1.016071429	2.02
AGAP008217	CYP6Z3	cytochrome P450 [Source:VB Community Annotation]	1.15	2.22	1.101071429	2.15
AGAP002863	COEAE6O	carboxylesterase alpha esterase [Source:VB Community Annotation]	1.09	2.13	1.016071429	2.02
AGAP002209	CYP325A2	cytochrome P450 [Source:VB Community Annotation]	0.91	1.88	1.086666667	2.12
AGAP002208	CYP325A3	cytochrome P450 [Source:VB Community Annotation]	0.87	1.83	1.034761905	2.05
AGAP002869	CYP6P2	cytochrome P450 [Source:VB Community Annotation]	0.86	1.82	1.0475	2.07
AGAP008356	CYP4H16	cytochrome P450 [Source:VB Community Annotation]	0.845	1.8	1.0625	2.09
AGAP002862	CYP6AA1	cytochrome P450 [Source:VB Community Annotation]	0.835	1.78	1.112857143	2.16

Supplementary Table 7: Cuticular genes. Top: Cuticular genes showing a mean or median fold change over 2. Headers show VectorBase ID, gene name, median log2 fold change, median fold change, mean log2 fold change and mean fold change. Bottom: Showing individual experiment log2 fold change.

GeneID	GeneName	median log2 Fold Change	median Fold Change	mean log2 Fold Change	mean Fold Change
AGAP008817	CPLCP3	2.56	5.9	1.989230769	3.97
AGAP006149	CPLCX3	2.25	4.76	1.440357143	2.71
AGAP010109	CPR150	2.18	4.53	2.305	4.94
AGAP000047	CPR130	2.05	4.14	1.461428571	2.75
AGAP006148	CPLCA3	2.03	4.08	1.960357143	3.89
AGAP008447	CPLCG4	1.825	3.54	2.014285714	4.04
AGAP006868	CPR140	1.795	3.47	1.328214286	2.51
AGAP006095	CPR106	1.75	3.36	1.546785714	2.92
AGAP005999	CPR24	1.6	3.03	1.474074074	2.78
AGAP009758	CPLCP11	1.54	2.91	1.4425	2.72
AGAP000820	CPR125	1.525	2.88	1.208571429	2.31
AGAP010124	CPR98	1.49	2.81	1.21047619	2.31
AGAP006848	CPR47	1.47	2.77	1.532307692	2.89
AGAP006829	CPR59	1.4	2.64	1.0675	2.1
AGAP006013	CPR33	1.3	2.46	1.4625	2.76
AGAP000988	CPAP3-A1c	1.19	2.28	0.721071429	1.65
AGAP009759	CPLCP12	1.165	2.24	1.207142857	2.31
AGAP006866	CPR63	1.16	2.23	1.083846154	2.12
AGAP008960	CPR110	1.065	2.09	0.99	1.99
AGAP005455	CPR14	0.95	1.93	1.222307692	2.33
AGAP006000	CPR25	0.8	1.74	1.022142857	2.03

GeneID	GeneName	Tiefora_v_Ngousso	Gou_v_Moz	Ban_v_BanS	BanRe_v_BanS	Bak_v_Kisumu	VK7_v_Kisumu	Cameroon_v_Ngousso	Chad_v_Ngousso	Niger_v_Ngousso	Nigeria_v_Ngousso
AGAP006149	CPLCX3	3.82	-1.42	2.35	0.25	-1.36	1.14	0.93	3.82	3.16	3.15
AGAP000989	CPAP3-A1a	2.23	1.62	0.35	-0.46	0.59	-0.61	0.65	1.31	2.04	1.77
AGAP000988	CPAP3-A1c	1.78	1.75	1.5	-0.87	-0.55	-1.7	-0.47	1.8	2.34	1.91
AGAP000047	CPR130	3.04	0.82	3.87	0.56	-1	-2.3	1.06	5	4.57	4.03
AGAP005459	CPR16	-0.38	1.2	0.22	-0.36	0.41	0.67	0.62	1.52	1.8	2.11
AGAP006001	CPR26	-1.69	0.29	-0.32	-0.25	-0.21	-3.16	-0.54	0.68	0.92	0.88
AGAP009758	CPLCP11	-2.08	0.56	1.47	0.52	0.23	0.08	-0.19	2.23	2.11	2.19
AGAP000820	CPR125	1.4	2.16	2.38	-0.1	-1.09	-1.89	-0.61	1.65	2.83	2.74
AGAP006261	CPR135	2.87	-0.11	1.8	0.51	0.24	-0.33	-1.61	-0.24	0.46	-0.47
AGAP005996	CPR21	0.66	0.95	1.52	0.1	0.58	0.27	0.26	1.12	1.07	1.24
AGAP006868	CPR140	1.76	2.01	1.59	-0.39	1.83	-0.55	-0.4	1.59	2.29	2.23
AGAP007980	CPCFC1	2.55	1.06	3.21	-0.29	-1.72	-0.89	-1	1.44	1.13	0.72
AGAP006829	CPR59	1.12	1.11	1.51	-0.48	-0.7	-2	-0.26	2.52	2.49	3.22
AGAP009877	CPR79	0.6	1	0.43	-0.65	0.62	0.32	-0.19	0.51	0.91	0.63
AGAP006931	CPR111	1.27	0.3	-0.29	-0.66	0.68	1.07	2.06	3.77	2.74	2.01
AGAP004690	CPF3	-0.86	1.36	3.87	2.82	0.9	-0.62	0.56	2.48	2.85	2.18
AGAP002613	CPR8	0.77	-0.53	0.54	-0.88	0.66	1	0.01	0.06	0.26	0.32
AGAP003379	CPR117	1.46	0.31	-0.36	0.62	0.18	1.14	0.93	2.95	3.69	4.84
AGAP008817	CPLCP3	2.79	2.9	3.6	0.91	-2.05	2.03	-0.76	2.54	2.58	0.17
AGAP009759	CPLCP12	0.28	0.4	1.47	0.62	0.57	-0.43	-0.07	2.68	3.09	3.3
AGAP006321	CPR71	2.49	0.52	2.73	-1.47	-2.32	-2.4	0.97			0.18
AGAP006148	CPLCA3	0.79	4.68	4.57	2.14	-1.27	2.39	-0.82	1.23	0.19	-2.1
AGAP000987	CPAP3-A1b	0.09	1.5	-0.27	-0.64	0.22	0.05	0.31	1.04	1.89	1.8
AGAP003375	CPR114	0.9	-0.67	-0.05	1	4.15	4.63	0.37	0.73	4.62	2.92
AGAP009878	CPR80	0.26	0.33	1.26	0.13	1.39	-0.66	1.9	1.81	2.05	2.29
AGAP003390	CPR124	0.02	0.36	-0.9	-0.06	-0.24	0.05	0.18	0.74	0.55	1.09
AGAP005455	CPR14		-3.18	4.53	0.23	1.34	-1.07	3.1	3.31	3.68	3.52
AGAP005999	CPR24	-0.45	1.6	4.36	-0.05	1.77	-1.95	1.36	4.1	4.22	3.61
AGAP006095	CPR106	-1.28	1.44	-1.65	-0.27	0.87	0.9	2.06	3.82	2.95	3.01
AGAP006836	CPR101		1.38	1.11	1.49	-0.36	-0.16		-0.42	0.94	
AGAP008960	CPR110	0.51	-3.02	0.08	-0.45	-0.81	-0.67	-0.6	2.26	1.71	0.99

GeneID	GeneName	Agboville_v_Mali	Agboville_v_Ngousso	Dabou_v_Mali	Dabou_v_Ngousso	Tiassale_v_Mali	Tiassale_v_Ngousso	Cam_fun_v_Fang	Fumoz_v_Fang	Ghana_fun_v_Fang	Malawi_fun_v_Fang
AGAP006149	CPLCX3	2.46	2.17	2.9	2.59	3.34	3.08	-0.8	0.41	-1.02	-1.24
AGAP000989	CPAP3-A1a	-0.39	-1.1	1.11	0.38	1.09	0.38	0.1	0.28	0.08	0.13
AGAP000988	CPAP3-A1c	0.7	1.71	1.72	2.72	0.88	1.89	0.2	0.36	0.41	0.34
AGAP000047	CPR130	2.8	3.73	3.57	4.49	3.09	4.04	-1.53	1.07	-0.58	-2.2
AGAP005459	CPR16	0.81	2.22	0.44	1.83	0.55	1.96	-0.15	-0.6	-0.48	-0.57
AGAP006001	CPR26	1.56	5.56	1.82	5.81	1.41	5.4	-0.48	0.7	1.06	0.54
AGAP009758	CPLCP11	2.55	1.78	2.95	2.2	3.06	2.32	1.18	1.61	0.67	0.93
AGAP000820	CPR125	1.98	2.08	3.12	3.2	3.67	3.8	-0.27	1.11	0.29	-0.46
AGAP006261	CPR135	2.31	2.75	1.98	2.42	1.81	2.26	-0.96	0.1	0.05	0.69
AGAP005996	CPR21	-0.36	0.97	-0.09	1.24	-0.5	0.84	0.41	0.82	-0.09	0.33
AGAP006868	CPR140	2.15	2.08	3	2.93	2.88	2.81	-0.18	0.71	0.31	-0.07
AGAP007980	CPCFC1	0.28	1.97	1.31	2.98	0.59	2.29	0.51	1.31	-0.15	-0.1
AGAP006829	CPR59	1.37	1.04	2.34	1.99	1.97	1.65	1.43	1.55	0.65	0.32
AGAP009877	CPR79	1.29	2.42	1.23	2.34	1.2	2.34	-1.38	-1.16	-1.35	-1.03
AGAP006931	CPR111	0.57	0.9	0.74	1.05	1.83	2.18	-0.96	1.19	-0.88	-0.44
AGAP004690	CPF3	-2.96	1.4	-4.11	0.2	-3.03	1.32	0.25	0.2	0.83	1.67
AGAP002613	CPR8	2.02	1.39	2.23	1.59	3.25	2.63	2.44	2.66	2.83	2.37
AGAP003379	CPR117	-2.38	3.82	-1.61	4.57	-1.81	4.39	0.36	0.13	1.57	0.98
AGAP008817	CPLCP3			0.68	0.69	0.85	0.87	3.83	4.88	4.12	4.13
AGAP009759	CPLCP12	1.42	1.94	2.26	2.77	2.35	2.88	-0.12	1.15	0.07	-0.34
AGAP006321	CPR71	0.59	0.03	2.67	2.11	2.68	2.12	2.16	2.44	3.17	3.19
AGAP006148	CPLCA3	2.26	2.8	3.59	4.12	4.22	4.8	1.86	2.95	1.92	1.79
AGAP000987	CPAP3-A1b	-0.31	0.17	-0.16	0.3	-0.04	0.44	0.17	0.76	0.13	0.74
AGAP003375	CPR114	1.53	0.25	1.96	0.73	1.13	-0.12	-0.48	-0.02	0.05	0.23
AGAP009878	CPR80	0.99	0.04	0.68	-0.27	1.28	0.35	-0.08	-0.57	0.29	0.12
AGAP003390	CPR124	0.58	0.38	0.98	0.77	0.6	0.4	-0.43	-0.28	0.41	0.57
AGAP005455	CPR14	0.59	0.61	2.93	2.93	1.14	1.16	-0.19	0.28	0.91	0.99
AGAP005999	CPR24	1.61	2.62	1.11	2.11	2.44	3.44	-1.15	-0.68	-0.67	0.14
AGAP006095	CPR106	3.32	-0.34	4.19	0.51	4.28	0.64	1.43	2.26	3.44	3.25
AGAP006836	CPR101	0.59	0.61	0.68	0.69	0.85	0.87	0.73	1.06	1.84	2.49
AGAP008960	CPR110	1.64	1.12	1.33	0.79	2.37	1.85	0.81	0.43	1.56	1.68

GeneID	GeneName	Uganda_fun_v_Fang	Asendabo_v_Moz	Chewaka_v_Moz	Tolay_v_Moz	Ethiopia_v_Dongola	Bouake_gamb_unexp_v_Kisumu	Bouake_colu_unexp_v_Ngousso	BusiaSurvivors_v_Kisumu
AGAP006149	CPLCX3	-1.3	6.35	4.81	7.08	1.24	2.33	-1.31	-8.6
AGAP000989	CPAP3-A1a	-0.31	3.44	1.92	4.2	0.97	0.15	-1.07	-1.45
AGAP000988	CPAP3-A1c	0.01	2.66	1.79	3.2	2.72	0.18	-3.42	-5.37
AGAP000047	CPR130	-1.57	4.45	3.36	4.65	1.3	-0.26	-0.95	-8.19
AGAP005459	CPR16	-0.63	0.86	0.47	1.52	1.64	0.28	-0.26	-1.57
AGAP006001	CPR26	0.04	1.39	1.04	2.03	2.23	0.08	-0.56	-2.68
AGAP009758	CPLCP11	0.32	5.56	4.27	6.47	2.02	0.46	-0.3	-4.78
AGAP000820	CPR125	-0.62	5.14	3.84	5.83	1.13	0.37	-1.6	-8.24
AGAP006261	CPR135	-0.01	1.71	0.28	3.23	5.2	0.65	-0.59	-4.91
AGAP005996	CPR21	0.08	0.68	0.53	0.7	0.63	-0.61	-0.19	0.26
AGAP006868	CPR140	0.08	4.01	2.98	4.31	2.2	1.28	0.16	-6.41
AGAP007980	CPCFC1	-0.85	6.35	4.67	6.89	-1.08	1.52	0.11	-8.69
AGAP006829	CPR59	0.16	4.28	2.49	4.69	1.5	0.75	-1.26	-5.56
AGAP009877	CPR79	-1.83	1.23	1.24	1.71	0.68	0.11	-0.21	0.77
AGAP006931	CPR111	0.02	2.55	2.52	2.42	2.87	0.06	-0.06	-1.63
AGAP004690	CPF3	0.43	3.61	1.92	4.54	-0.64	-1.19	2.11	-4.64
AGAP002613	CPR8	2.38	-0.01	-0.42	0.61	-1.7	0.75	-0.99	-1.33
AGAP003379	CPR117	0.48	0.69	-1.37	1.15	-0.7	1.03	-1.34	-0.42
AGAP008817	CPLCP3	4.86	2.75	2.82	2.67	4	1.8	-0.88	-1.06
AGAP009759	CPLCP12	-0.63	4.99	4.18	5.81	0.3	1.18	-1.08	-7.24
AGAP006321	CPR71	3.21		0.77		0.51	1.03	-1.29	-4.53
AGAP006148	CPLCA3	1.5	5.75	4	6.64	1.59	0.3	-0.17	-6.83
AGAP000987	CPAP3-A1b	0.26	-0.04	0.04	0.23	1.92	0.69	-1.01	-0.35
AGAP003375	CPR114	0.13	1.19	0.85	-0.92	0.54	-0.74	-1.45	1.07
AGAP009878	CPR80	-0.16	0.08	0.04	0.56	-0.39	-0.66	-0.76	0.42
AGAP003390	CPR124	0.5	0.98	0.89	0.68	0.91	-0.31	0.13	-0.82
AGAP005455	CPR14	-0.19	0.76	1.56		-0.39	2.41	0.19	0.63
AGAP005999	CPR24	-0.74	4.03	2.6	5.22		1.56	0.08	-2.49
AGAP006095	CPR106	2.95	2.06	1.11	3.37	2.31	-0.73	-1.1	-1.49
AGAP006836	CPR101	0.9	0.76	0.77	0.92	1.51	-1.4	-0.9	-2.77
AGAP008960	CPR110	1.17	4.63	4.87	5.1	0.52	1.01	1.33	-4.49

Supplementary Text 1

We used RNA-Seq data from Nagi et al. (2023) to compare different read counting methods. The dataset consisted of RNA-Seq samples from both insecticide-susceptible (Kisumu strain, n=4) and insecticide-resistant (G28-Busia survivors, n=4) *Anopheles gambiae* mosquitoes.

Read Counting Method Comparison

We compared three commonly used read counting methods:

1. HISAT2-featureCounts: RNA-Seq reads were aligned to the Anopheles gambiae PEST reference genome (AgamP4.12) using HISAT2 v2.2.1 with default parameters. The resulting BAM files were processed with featureCounts (from the Subread package v2.0.1) to quantify reads mapping to annotated genes using the AgamP4.12 gene annotation file.

2. HISAT2-htseq-count: The same HISAT2-aligned BAM files were processed using htseq-count v0.13.5 with parameters `-f bam -r pos -s no -t exon -i Parent` to quantify reads per gene.

3. Kallisto: Raw RNA-Seq reads were processed with Kallisto v0.46.1 using a transcriptome index built from AgamP4.12 transcripts. Quantification was performed with parameter `-b 100` for 100 bootstrap samples.

For each method, we generated count matrices that were subsequently analyzed using DESeq2 v1.26.0 in R v3.6.3 to perform differential expression analysis between resistant and susceptible samples. We then calculated Pearson correlation coefficients between the log2 fold-change values obtained from each method.

```bash script to perform counting
#!/bin/bash

# Set reference files
GFF_FILE="resources/reference/Anopheles-gambiae-PEST_BASEFEATURES_AgamP4.12.gff3"
KALLISTO_INDEX="resources/reference/kallisto.idx"

# Sample names
SAMPLES=("Kisumu1" "Kisumu2" "Kisumu3" "Kisumu4" "G28-BusiaSurvivors1" "G28-BusiaSurvivors2"
"G28-BusiaSurvivors3" "G28-BusiaSurvivors4")

# Loop through each sample and run the tools
for SAMPLE in "${SAMPLES[@]}"
do
    BAM_FILE="results/alignments/${SAMPLE}.hisat2.bam"
    R1_FASTQ="resources/reads/${SAMPLE}_1.fastq"
    R2_FASTQ="resources/reads/${SAMPLE}_2.fastq"

    FEATURECOUNTS_OUTPUT="${SAMPLE}_featureCounts_output.txt"
    HTSEQCOUNT_OUTPUT="${SAMPLE}_htseq_counts_output.txt"
    KALLISTO_OUTPUT="results/counts/${SAMPLE}"
```

```
    # Run featureCounts
    echo "Running featureCounts for ${SAMPLE}..."
    featureCounts -a $GFF_FILE -o $FEATURECOUNTS_OUTPUT -p -B -t exon -g Parent -C $BAM_FILE
    if [ $? -ne 0 ]; then
        echo "featureCounts failed for ${SAMPLE}"
        exit 1
    fi

    # Run htseq-count
    echo "Running htseq-count for ${SAMPLE}..."
    htseq-count -f bam -r pos -s no -t exon -i Parent $BAM_FILE $GFF_FILE > $HTSEQCOUNT_OUTPUT
    if [ $? -ne 0 ]; then
        echo "htseq-count failed for ${SAMPLE}"
        exit 1
    fi

    # Run kallisto
    echo "Running kallisto for ${SAMPLE}..."
    mkdir -p $KALLISTO_OUTPUT
    kallisto quant -i $KALLISTO_INDEX -o $KALLISTO_OUTPUT -b 100 -t 24 $R1_FASTQ $R2_FASTQ 2> "logs/kallisto/quant_${SAMPLE}.log"
    if [ $? -ne 0 ]; then
        echo "kallisto failed for ${SAMPLE}"
        exit 1
    fi

done

echo "All tools have completed successfully for all samples."
```

Fragment vs Read Count Comparison
To evaluate the impact of fragment-based versus read-based counting approaches, we processed the same dataset using both methodologies:

Fragment counting: We used featureCounts with parameters -p --countReadPairs -B -C -t exon -g Parent to count fragments (paired-end reads) rather than individual reads. The --countReadPairs option ensures that each fragment (read pair) is counted once, while -B requires both reads in a pair to be aligned, and -C excludes chimeric fragments.

Read counting: We used featureCounts with parameters -p -t exon -g Parent -C to count individual reads. In this approach, each read that maps to a feature is counted separately, even if both reads from a pair map to the same feature.

For both counting methods, we used the Anopheles gambiae PEST reference annotation (AgamP4.12.gff3) with -t exon to count at the exon level and -g Parent to aggregate counts to the gene level. The resulting count matrices were then analyzed using DESeq2 with identical parameters to perform differential expression analysis.

For both comparisons (read counting methods and fragment vs. read counting), we used the following metrics to assess concordance:

1. Correlation of raw counts
2. Correlation of normalized counts (after DESeq2 normalization)
3. Correlation of log2 fold-change values from differential expression analysis
4. Consistency of differential expression calls (genes identified as significantly differentially expressed)

```
#!/bin/bash

# Set reference files
GFF_FILE="resources/reference/Anopheles-gambiae-PEST_BASEFEATURES_AgamP4.12.gff3"

# Sample names
SAMPLES=("Kisumu1" "Kisumu2" "Kisumu3" "Kisumu4" "G28-BusiaSurvivors1" "G28-BusiaSurvivors2"
"G28-BusiaSurvivors3" "G28-BusiaSurvivors4")

# Max number of parallel processes
MAX_JOBS=4

# Function to process a sample
process_sample() {
    SAMPLE=$1
    BAM_FILE="results/alignments/${SAMPLE}.hisat2.bam"

    FEATURECOUNTS_FRAGMENTS="${SAMPLE}_featureCounts_fragments.txt"
    FEATURECOUNTS_READS="${SAMPLE}_featureCounts_reads.txt"
```

```
    echo "Processing ${SAMPLE}..."

    # Run featureCounts in fragment mode (counting read pairs)
    echo "Running featureCounts in fragment mode for ${SAMPLE}..."
    featureCounts -a $GFF_FILE -o $FEATURECOUNTS_FRAGMENTS -p --countReadPairs -B -t exon -g
Parent -C $BAM_FILE
    if [ $? -ne 0 ]; then
        echo "featureCounts fragment mode failed for ${SAMPLE}"
        return 1
    fi

    # Run featureCounts in read mode (counting individual reads)
    echo "Running featureCounts in read mode for ${SAMPLE}..."
    featureCounts -a $GFF_FILE -o $FEATURECOUNTS_READS -p -t exon -g Parent -C $BAM_FILE
    if [ $? -ne 0 ]; then
        echo "featureCounts read mode failed for ${SAMPLE}"
        return 1
    fi

    echo "Completed both counting methods for ${SAMPLE}"
    return 0
}

# Array to store background process IDs
pids=()

# Process each sample
for SAMPLE in "${SAMPLES[@]}"; do
    # Wait if we've reached the maximum number of parallel jobs
    while [ ${#pids[@]} -ge $MAX_JOBS ]; do
        # Check which processes have finished and remove them from the array
        for i in "${!pids[@]}"; do
            if ! kill -0 ${pids[$i]} 2>/dev/null; then
                unset pids[$i]
            fi
        done
        # Reindex the array to remove gaps
        pids=("${pids[@]}")
        # Sleep briefly before checking again
        sleep 1
    done

    # Process the sample in the background
    process_sample "$SAMPLE" &
    # Store the process ID
    pids+=($!)
done
```

```
# Wait for all remaining background processes to complete
for pid in "${pids[@]}"; do
    wait $pid
    if [ $? -ne 0 ]; then
        echo "Error occurred in one of the background processes"
        exit 1
    fi
done

echo "All counting completed successfully for all samples."
```

Supplementary Text 2

H12 is calculated in stepping windows across the genome. Calibration is first performed on each cohort, to determine the optimal window size. H12 is run at a variety of window sizes, and the smallest window size in which > 95% of windows have a H12 value below 0.1 is chosen as the window size for that cohort.

Signals are then defined by fitting an exponential model to the data moving along the genome in windows, using the lmfit package in python. This is performed on the real H12 data, as well as a dataset in which the H12 value is constant. The difference between the Akaike Information Criterion (AIC) of the null and H12 model, provides a summary statistic, called Delta_I of how strong the signal is. Overlapping signals are de-duplicated, with the highest Delta_I peak retained.

Parameters of the exponential model, include skew, decay, and the center of the peak. We define the focus of the selection signal, to be:

focus_right = center + .25 * (2**(-skew_param) * decay_param)
focus_left = center - .25 * (2**skew_param * decay_param)

This approach was developed and coded by Alistair Miles for the *Anopheles* selection-atlas (in preparation).
