# Supplementary material for: A multi-omic meta-analysis reveals novel mechanisms of insecticide resistance in malaria vectors
Source: Commun Biol. 2025 May 23;8:790. doi: 10.1038/s42003-025-08221-6 (PMC12102355; doi:10.1038/s42003-025-08221-6)
Supplement: Supplementary file 5 — Description of Additional Supplementary Files [file 42003_2025_8221_MOESM5_ESM.docx]

Description of Additional Supplementary Files

**File name:** Supplementary Data 1

**Description:** Mean fold change across all experiments for each gene in the analysis. VectorBase ID, Gene Name, Gene Description and both log2 and raw mean fold change.

**File name:** Supplementary Data 2

**Description:** Median fold change across all experiments for each gene in the analysis. VectorBase ID, Gene Name, Gene Description and both log2 and raw median fold change.

**File name:** Supplementary Data 3

**Description:** GENIE3 output for transcription factor interactors. VectorBase GeneID, GENIE3 Score, gene name and description, transcription factor name and transcription factor ID.
